# EBP50 regulates the apoptosis of pancreatic cancer cells by decreasing the expression levels of Bcl-2

**DOI:** 10.3892/etm.2014.1831

**Published:** 2014-07-07

**Authors:** MENGYAO JI, LEI YUAN, XIAOGUANG LV, WEIGUO DONG, XIULAN PENG

**Affiliations:** 1 Department of Gastroenterology, Renmin Hospital of Wuhan University, Wuhan, Hubei 430060, P.R. China; 2 Criminal Science and Technology Studio, Chongqing Banan Police Security Bureau, Chongqing, Chongqing 401320, P.R. China; 3 Department of Oncology, The Fifth Hospital of Wuhan, Wuhan 430050, P.R. China

**Keywords:** ezrin-radixin-moesin-binding protein 50, overexpression, pancreatic cancer, B-cell lymphoma 2

## Abstract

Increasing evidence has demonstrated that ezrin-radixin-moesin (ERM)-binding phosphoprotein 50 (EBP50) is involved in the malignant transformation of numerous human cancers. The present study investigated the involvement of EBP50 overexpression in the tumorigenicity of pancreatic cancer (PC). The results revealed that overexpression of EBP50 suppressed cell growth, promoted cell apoptosis and arrested G1-to-S phase progression in two human PC cell lines. Overexpression of EBP50 also suppressed B-cell lymphoma 2 (Bcl-2) expression. Furthermore, nude mouse tumor xenograft models were established by the subcutaneous injection of cell lines stably transfected with an EBP50-expressing plasmid. The *in vivo* data indicated that overexpression of EBP50 inhibited the growth of the PC tumors and induced cell apoptosis. Thus, the present study demonstrated that EBP50 overexpression induces growth inhibition and apoptosis in PC by decreasing Bcl-2 expression. The results suggest that EBP50 may function as a potential tumor suppressor *in vivo* and *in vitro*.

## Introduction

Pancreatic cancer (PC) has one of poorest prognosis rates of all tumors and no successful cure has yet been identified. Despite impressive improvements in surgical and chemotherapeutic approaches (1), the 5-year survival rate remains <5% (2). Thus, it is urgently necessary to search for novel highly specific and sensitive treatments in order to improve the prognosis of patients with PC.

Ezrin-radixin-moesin (ERM)-binding phosphoprotein 50 (EBP50), also termed Na^+^/H^+^ exchanger regulatory factor 1 (NHERF1), belongs to the NHERF protein family. This family contains important molecular scaffold proteins that coordinate a diverse range of regulatory processes for ion transport and second messenger cascades (3–5). EBP50 comprises two tandem PSD-95/discs large/ZO-1 (PDZ) domains and a carboxyl (C)-terminal EB region (3,6,7), which is a 50-kD microvillar scaffolding protein. EBP50 binds with the majority of protein receptors, including the parathyroid hormone type 1, β2-adrenergic, κ-opioid, parathyroid hormone type 1 and G protein-coupled receptors, through the first PDZ domain (8–10). Growth factor tyrosine kinase receptors, including the platelet-derived and epidermal growth factor receptors, are also able to interact with EBP50 (11–13). The second PDZ domain is able to bind with only a few proteins, such as β-catenin, sodium-hydrogen exchanger 3 (NHE3) and yes-associated protein 65 (Yap 65) (14,15).

A previous study revealed that the downregulation of EBP50 promoted PC cell proliferation, increased the colony-forming ability of cells and accelerated G1-to-S phase progression (16). However, to the best of our knowledge, no study on the effect of upregulation of EBP50 on PC cell lines has been published. To examine whether EBP50 overexpression was efficient at treating PC, the present study used the Pbk-CMV-HA-EBP50 plasmid to upregulate EBP50 expression in PC cells and investigate its effect on SW1990 and PANC-1 PC cells. Furthermore, the antitumor efficacy of Pbk-CMV-HA-EBP50 was detected *in vivo,* using two mouse tumor xenograft models, individually originating from PANC-1 and SW1990 cells.

## Materials and methods

### Cell culture and transfection

Human pancreatic cancer cell lines PANC-1 and SW1990 were purchased from the Cell Bank of the Shanghai Institutes for Biological Sciences (Shanghai, China). They were maintained in a laboratory and cultured in HyClone™ RPMI-1640 medium (Gibco-BRL, Grand Island, NY, USA) containing 10% fetal calf serum, 100 U/ml penicillin and 100 μg/ml streptomycin at 37°C and 5% CO_2_ in a Forma™ incubator ( Hera Cell, Thermo Scientific, Waltham, MA, USA). The Pbk-CMV-HA-EBP50 plasmid was kindly provided by Dr. Randy Hall from Emory University (Atlanta, GA, USA) and Pbk-CMV-HA was obtained from Santa Cruz Biotechnology, Inc. (Santa Cruz, CA, USA). Cells were seeded on a six-well plate at 2×10^5^/ml and transfected with equal amounts of Pbk-CMV-HA-EBP50 to generate EBP50-PANC-1 and EBP50-SW1990 cells, or Pbk-CMV-HA to create HA-PANC-1 and HA-SW1990 cells. The transfection was conducted using Lipofectamine^®^2000 (Invitrogen Life Technologies, Carlsbad, CA, USA) in accordance with the manufacturers’ instructions. The cells were collected after 24 or 48 h. Transfection with Pbk-CMV-HA was used as the negative control (17). The stably transfected cell lines were constructed using a previously described method (16). Cells were trypsinized and reseeded into a 12-well plate. Stably transfected cell clones were selected using G418 solution (Gibco-BRL). Once the single-cell clones were isolated, the clones were expanded. Western blot analysis was carried out to determine the transfection efficiency of each cell clone.

### Western blot analysis

The cultured cells were collected and lysed using radioimmunoprecipitation (RIPA, Thermo Scientific) lysis buffer. The protein concentration was measured using a bicinchoninic acid (BCA) Protein Assay kit (Pierce Biotechnology, Inc., Rockford, IL, USA). Equal amounts of proteins were separated on sodium dodecyl sulfate polyacrylamide gel electrophoresis (SDS-PAGE) gels and electro-transferred to nitrocellulose membranes at 4°C for 2 h. The primary antibody was rabbit polyclonal anti-human EBP50 (Novus, Saint Charles, MO, USA), which was added to the proteins at 1:800 dilution and incubated overnight at 4°C. Another antibody was anti-Bcl-2 (1:1,000; Abcam, Cambridge, MA, USA), which was also incubated overnight at 4°C. The proteins were then incubated with horseradish peroxidase-conjugated anti-rabbit antibodies (Sigma, St. Louis, MO, USA) for 1 h at 4°C. The proteins were subsequently detected by enhanced chemiluminescence (ECL; Amersham Pharmacia Biotech, Piscataway, NJ, USA) according to the manufacturer’s instructions and quantified by densitometry using UN-SCAN-IT software (Silk Scientific Corp., Orem, UT, USA) (18) with β-actin used as an internal control.

### Cell proliferation assay

A cell counting kit-8 (CCK-8; Dojindo, Kumamoto, Japan) colorimetric assay was used to determine cell proliferation and viability. Cells were washed twice with ice-cold phosphate-buffered saline (PBS), harvested by trypsinization, counted and plated at a final density of 5×10^3^ cells/well in a 96-well plate. Cell viability was assessed once daily for seven consecutive days using the CCK-8. The absorbance was detected in a microplate reader model 450 (Bio-Rad Laboratories, Hercules, CA, USA) at 450 nm wavelength. Flow cytometry (FACSCalibur, Becton-Dickinson, Franklin Lanes, NJ, USA) was used to analyze the cell cycle of the stained cells (data not shown) and the cell population in each phase was calculated by computer model fitting (Verity Software House, Topsham, ME, USA).

### Cell cycle analysis by Annexin V-fluorescein isothiocyanate (FITC) staining

The treated cells were plated at a density of 1×10^6^ cells/ml in six-well plates. The culture medium was substituted with fresh medium. The cells were collected, washed with cold PBS and suspended in 1× binding buffer at 10^5^–10^6^ cells/ml. Annexin V and propidium iodide (PI) were added to the prepared cell suspension. The cell suspension was kept on ice and incubated for 10 min in the dark. It was subsequently diluted with 1× binding buffer. Flow cytometry was used to analyze the cell cycle of the stained cells (19).

### Hoechst 33258 staining

Stably transfected cells were fixed, stained with Hoechst 33258 and observed using a fluorescence microscope (model IX71, Olympus, Tokyo, Japan). The stained cells were identified as apoptotic if they were highly condensed with brightly stained nuclei, or non-apoptotic if they were stained pale blue (20).

### Tumor xenografts

A total of 30 female BALB/c nude mice aged 6–8 weeks were purchased from Beijing HFK Bioscience Co., Ltd. (Beijing, China) and were kept under specific pathogen-free conditions. The mice were randomly divided into six groups (n=5) and subcutaneously injected with cells of one of the following types: i) PANC-1; ii) HA-PANC-1; iii) EBP50-PANC-1; iv) SW1990; v) HA-SW1990; and vi) EBP50-SW1990. After 36 days, the mice were sacrificed and the tumor tissues were analyzed by hematoxylin and eosin (H&E) staining, immunohistochemical analysis and a terminal deoxynucleotidyl transferase dUTP nick end labeling (TUNEL) assay (18,21).

The tissues were harvested and fixed in 4% paraformaldehyde at 4°C. Samples were embedded in paraffin, sectioned at 5-μm thickness with a microtome and stained with H&E staining for light microscopic examination (18).

Immunohistochemistry staining was performed on paraffin sections with anti-EBP50 polyclonal rabbit antibody (1:800 dilution, Novus, Saint Charles, MO, USA) according to the manufacturer’s protocol and included the following brief steps: Deparaffinization followed by antigen retrieval, initial blocking followed by incubation with the primary antibody, and further washing and blocking followed by incubation with the biotinylated secondary antibody. Finally, the sections were washed, blocked again, washed, mounted and observed. Negative controls were achieved by substituting the primary antibody with an isotype-matched irrelevant antibody (21).

The TUNEL assay was performed on the paraffin-embedded sections using a standard TUNEL assay kit (Roche, Mannheim, Germany). Using the same method as the immunohistochemical analysis, the sections were treated (n=5, in each group) in order to prepare them at 24 h after reperfusion. The staining was performed according to the manufacturer’s instructions. The nucleus of the cells that was stained brown was an indicator of the presence of TUNEL-positive cells.

The animal experiments were approved by the Institutional Animal Care and Use Committee of Wuhan University (Wuhan, Hubei).

### Statistical analyses

Data are presented as mean ± standard deviation. Analysis of variance (ANOVA) was used to perform statistical comparisons of *in vitro* and *in vivo* data. P<0.05 was considered to indicate a statistically significant difference.

## Results

### Overexpression of EBP50 inhibits the proliferation of human PC cells

**Figure 1:**
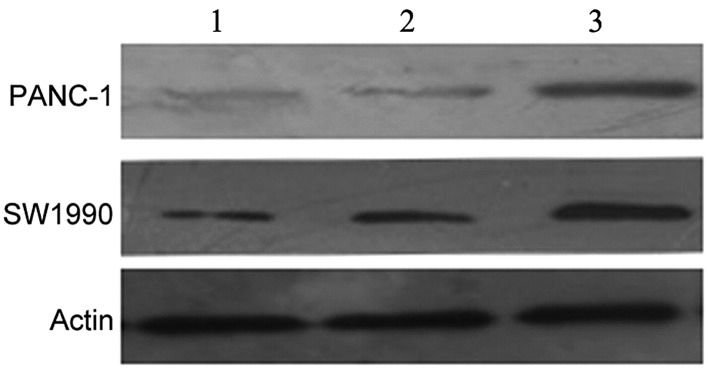
PANC-1 and SW1990 cells clones with a stable overexpression of ezrin-radixin-moesin (ERM)-binding phosphoprotein 50 (EBP50) were established. All experiments were performed in triplicate.

**Figure 2:**
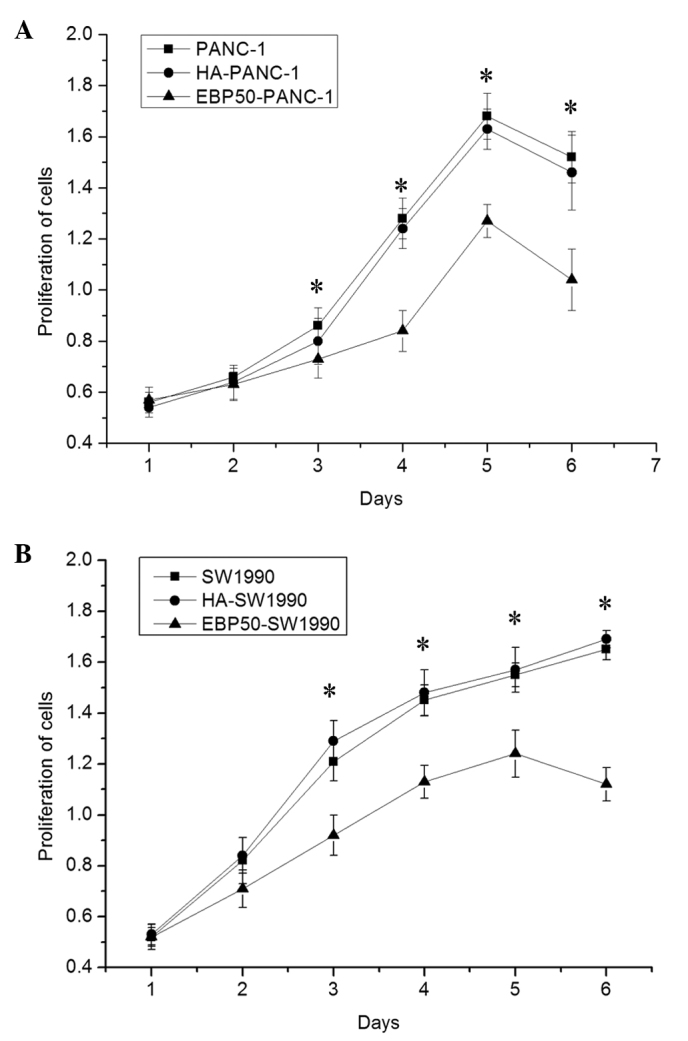
Cell counting kit-8 (CCK-8) assay of cell growth in two human pancreatic cancer cell lines following ezrin-radixin-moesin (ERM) binding phosphoprotein 50 (EBP50) overexpression. Absorbance values were measured at 450 nm with a microplate reader. (A) PANC-1 cell groups; (B) SW1990 cell groups. All experiments were performed in triplicate. Data are presented as mean ± standard deviation. ^*^Significantly different from the corresponding control group (P<0.05).

The Pbk-CMV-HA-EBP50 plasmid was stably transfected into PANC-1 and SW1990 cells. Following selection with G418 solution, western blot analysis was carried out to identify the stable clones. The data revealed that the expression levels of the EBP50 protein were significantly higher in EBP50-PANC-1 and EBP50-SW1990 cells than those in the PANC-1/HA-PANC-1 and SW1990/HA-SW1990 cells, respectively (Fig. 1), thus indicating that the EBP50 protein was upregulated in the two PC cell lines. Subsequently, to test the effect of EBP50 overexpression on the suppression of PC cell proliferation, a CCK-8 assay was performed to detect the cell viability of two treated human PC cell lines. There was significant suppression of PC cell proliferation in the EBP5-PANC-1 and EBP5-SW1990 cells; however, there was no significant difference in proliferation between the untreated and negative control groups in the two PC cell lines (Fig. 2).

### EBP50 overexpression induces G1-to-S phase cell cycle arrest and cell apoptosis in human PC cells

**Figure 3:**
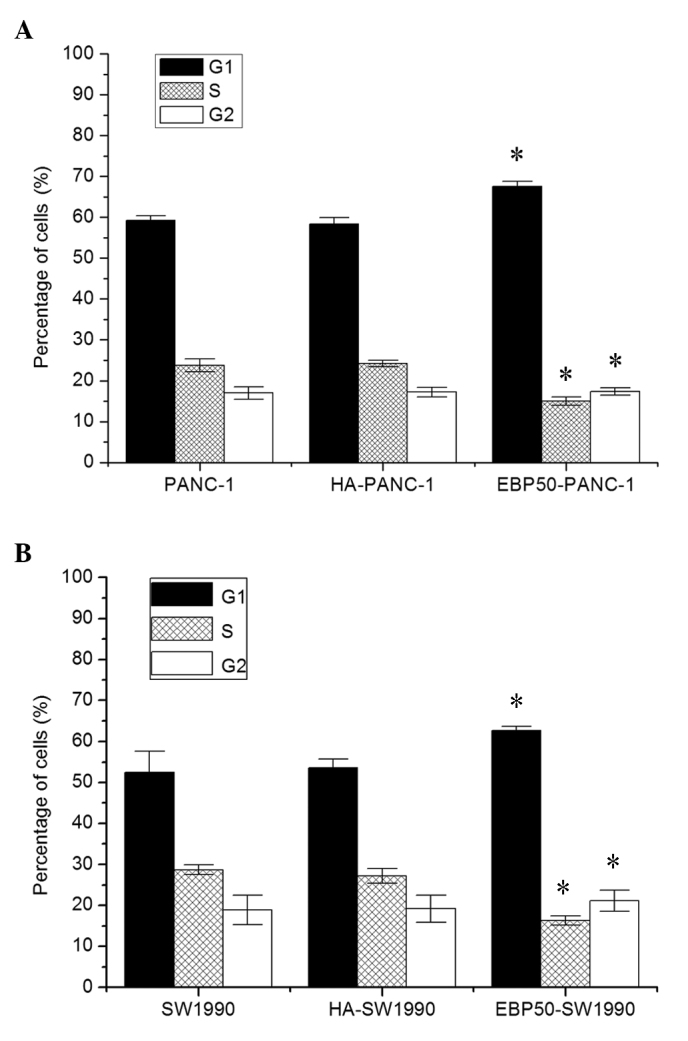
Impact of ezrin-radixin-moesin (ERM)-binding phosphoprotein 50 (EBP50) overexpression on the regulation of the cell cycle in two pancreatic cancer cell lines analyzed by fluorescence-activated cell sorting (FACS) flow cytometry. (A) PANC-1 cell groups. (B) SW1990 cell groups. All experiments were completed in triplicate. Data are presented as mean ± standard deviation. ^*^Significantly different from the corresponding control group (P<0.05).

To examine the effect of EBP50 overexpression on the cell cycle, flow cytometry was carried out to detect the cell cycle stages of two PC cell lines following stable transfection. All lines transfected with Pbk-CMV-HA exhibited a pattern of phase distribution similar to that of the corresponding untransfected cell lines. However, the phase distribution of the two PC cell lines transfected with Pbk-CMV-HA-EBP50 was markedly different from that of the control and untreated groups. There was a significant increase in the percentage of G0/G1 phase cells and a marked reduction in the percentage of S phase cells in the Pbk-CMV-HA-EBP50 groups compared with the corresponding control groups (Fig. 3). Thus, EBP50 overexpression may promote cell apoptosis through arresting cell cycle progression of the two PC cell lines between the G1 and S phases.

**Figure 4:**
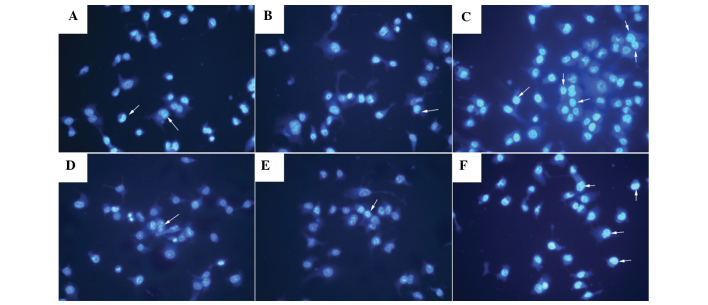
Apoptosis detection in two pancreatic cancer cell lines analyzed by Hoechst 33258 staining. (A-C) Representative data of PANC-1 cells: (A) PANC-1; (B) HA-PANC-1 and; (C) ezrin-radixin-moesin (ERM)-binding phosphoprotein 50 (EBP50)-PANC-1 cells. (D-E) Representative data of SW1990 cells: (D) SW1990; (F) HA-SW1990 and; (F) EBP50-SW1990 cells. Magnification, ×200.

To further investigate the role of EBP50 overexpression in the regulation of cell apoptosis, a Hoechst 33258 stain was used to detect the levels of cell apoptosis in the two PC cell lines following stable transfection. As shown in Fig. 4, the apoptosis rates of cells that were stably transfected with Pbk-CMV-HA-EBP50 were markedly higher compared with those of the cells in the corresponding untreated and control groups. These data further support the hypothesis that EBP50 overexpression promotes apoptosis in PC cells.

### EBP50 overexpression promotes the apoptosis rate of PC cells by altering the expression level of the Bcl-2 protein

**Figure 5:**
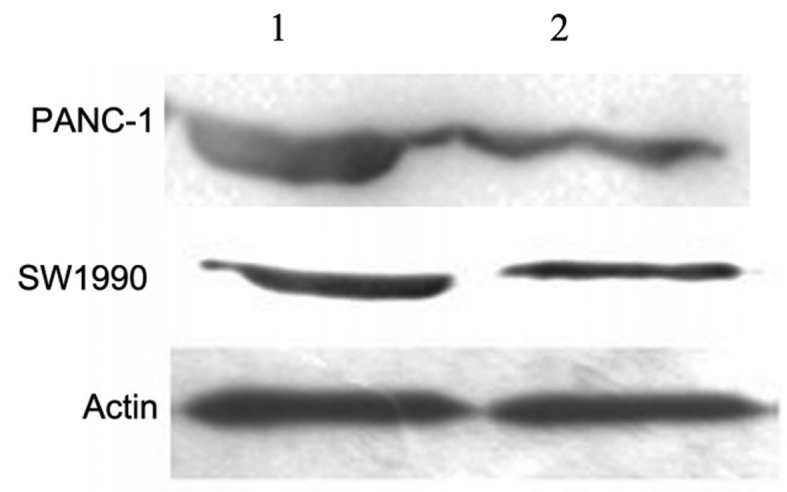
Western blot analysis of B-cell lymphoma 2 (Bcl-2) protein expression in two human pancreatic cancer cell lines. Proteins were detected by enhanced chemiluminescence with β-actin serving as a loading control.

To explore the molecular mechanisms underlying the anticancer effect of Pbk-CMV-HA-EBP50, western blot analysis was carried out to determine the expression levels of Bcl-2, an anti-apoptosis protein, in the PC cells. The results revealed that Bcl-2 was markedly suppressed in the two PC cell lines when EBP50 was overexpressed (Fig. 5). These data suggest that the anticancer effects observed during EBP50 overexpression occur due to the suppression of Bcl-2 expression.

### EBP50 overexpression inhibits the growth of PC cells and promotes cell apoptosis in vivo

**Figure 6:**
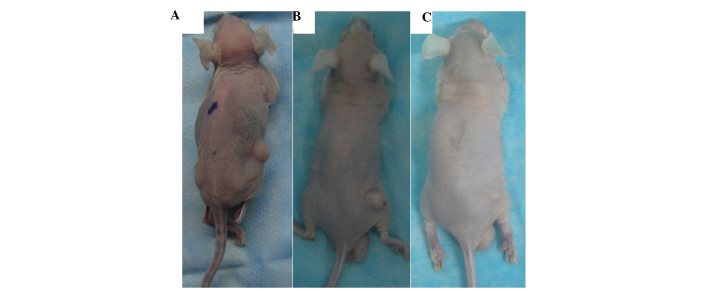
Ezrin-radixin-moesin (ERM)-binding phosphoprotein 50 (EBP50) overexpression inhibited the growth of pancreatic cancer cells *in vivo*. Nude mice were injected with (A) EBP50-PANC-1, (B) SW1990 and (C) HA-SW1990.

To detect the therapeutic efficacy of EBP50 overexpression *in vivo*, mice were randomly divided into six groups. Six tumor xenograft nude mice models were established by subcutaneously injecting six types of PC cells. After 36 days, the tumors in the EBP50-PANC-1-treated mice were significantly smaller compared with those in the HA-PANC-1-treated mice and PANC-1-treated mice (all P<0.01); however, the difference in tumor sizes between the HA-PANC-1 and PANC-1 groups was not statistically significant (Fig. 6). Similarly, the tumor sizes of the EBP50-SW1990-treated mice were smaller than the tumors in the corresponding control and untreated groups. The tumor sizes of mice in the SW-1990 untreated and control groups were similar. The *in vivo* results of the present study indicate that EBP50 overexpression was able to inhibit the growth of PC tumors.

**Figure 7:**
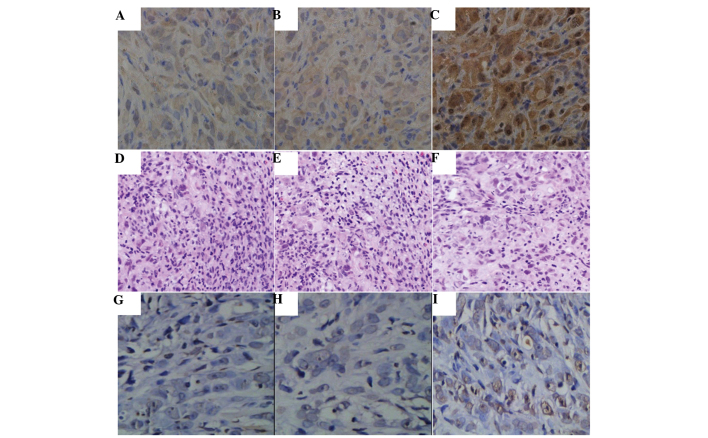
Representative images from the PANC-1 cell group of nude mice that were subcutaneously injected with three cell lines. Immunohistochemical staining of ezrin-radixin-moesin (ERM)-binding phosphoprotein 50 (EBP50) in the xenograft tissue sections of nude mice subcutaneously injected with (A) PANC-1, (B) HA-PANC-1 and (C) EBP50-PANC-1. Hematoxylin and eosin staining of the xenograft tissue sections of nude mice subcutaneously injected with (D) PANC-1, (E) HA-PANC-1 and (F) EBP50-PANC-1. Terminal deoxynucleotidyl transferase dUTP nick end labeling (TUNEL) staining of the xenograft tissue sections of nude mice subcutaneously injected with (G) PANC-1, (H) HA-PANC-1 and (I) EBP50-PANC-1. For D-F, magnification is ×100; for A–B and G-I, magnification is ×400.

Immunohistochemical analysis revealed that the expression levels of EBP50 were significantly higher in the EBP50-PANC-1 cell tumors compared with those in the tumors treated with negative control (HA-PANC-1) or untreated cells (PANC-1) (Fig. 7). H&E staining results demonstrated that there was a compact mass of epithelial cells in the untreated and control group tumors (Fig. 7). However, the EBP50-PANC-1 cell tumors appeared as loose epithelial cells with scattered apoptotic cells characterized by dark shrunken cytoplasms and purple pyknotic nuclei. The TUNEL assay data revealed that tumors in which EBP50 was overexpressed had a significantly higher apoptosis rate compared with that of tumors that were treated with the negative control or untreated cells (Fig. 7), indicating that EBP50 overexpression promoted apoptosis *in vivo*.

## Discussion

The current study revealed that EBP50 exhibits significant antitumor effects in PC cells *in vitro* and *in vivo*. The data demonstrated that EBP50 inhibited the proliferation of PC cells and promoted cell apoptosis, altered cell cycle progression, suppressed the growth of mouse xenograft tumors and promoted their apoptosis. In tumors with EBP50 overexpression, the expression levels of Bcl-2 were markedly reduced, consistent with the presumed mechanism of action of EBP50. The current study provides the first evidence, to the best of our knowledge, to suggest that EBP50 overexpression may suppress the tumorigenicity of PC *in vivo* and *in vitro* by decreasing the expression levels of Bcl-2.

A previous study reported that the downregulation of EBP50 promoted PC cell proliferation, increased the colony-forming ability of cells and accelerated G1-to-S phase progression (16). In the present study, EBP50 overexpression significantly induced growth inhibition, G1-to-S cell cycle arrest and cell apoptosis in two PC cell lines. Furthermore, it was revealed that the expression levels of Bcl-2 were significantly reduced in the cells with EBP50 overexpression. The marked effect of EBP50 overexpression *in vitro* prompted further study to detect its effect *in vivo*. It was demonstrated that the overexpression of EBP50 exhibited a notable effect on growth inhibition and apoptosis in EBP50-PANC-1 and EBP50-SW1990 tumors through a reduction in the expression levels of Bcl-2.

The expression of EBP50 (3,6,8) has been reported in a number of human tumors, including breast and liver cancers. The majority of these studies have demonstrated that EBP50 may have an anticancer effect in human cancers. One study revealed that EBP50 was able to interact with phosphatase and tensin homolog (PTEN) to exert an inhibitory effect on the phosphoinositide-3 kinase (PI3K)/Akt pathway (22). Another study demonstrated that overexpression of EBP50 decreased the colony-formation ability of cells and inhibited cell proliferation through the suppression of extracellular-signal-regulated kinase (ERK) activity (23). The binding of EBP50 to the epidermal growth factor receptor (EGFR) and neurofibromin 2 (NF2) may also result in tumor suppression (24). EBP50 may also form ternary complexes with platelet-derived growth factor (PDGF) and NF2 to generate anticancer effects (25). The results of the current study are consistent with published data on the role of EBP50 in breast tumors (6).

The present study indicated that high expression levels of EBP50 are associated with a lower malignant potential of PC tumors. Thus, it is proposed that EBP50 expression may be a valid method of combating PC.
